# The complete mitochondrial genome of the strawberry aphid *Chaetosiphon fragaefolii* Cockerell, 1901 (Hemiptera: Aphididae) from California, USA

**DOI:** 10.1080/23802359.2021.1915206

**Published:** 2021-07-19

**Authors:** Miguel Acosta, Diana Alcantar, Ivan Alier-Reyes, Carlos Alvarez, Crystal B. Arroyo, David Calderon, David Cardenas, Alejandro R. Castro, Janelle K. Companion, Cristian Constante, Evelyn S. Diaz Telles, Gabriel Fletes, Fatima C. Gama, Celia Garcia Perez, Abigail Garcia, Bailey Garcia, Brandon S. Gutierrez, Karina L. Guzman, Cecilia Hernandez, Jeffery R. Hughey, Monica Ibarra Flores, Adilene I. Jacobo, Brianna Lopez, Norma C. Lopez-De Leon, Jaden D. Martinez, Nayelli Mendoza, Kimberly Perez, Lucio J. Perez, Milagros Perez-Moreno, Caitlin D. Pineda, Elizabeth Pinedo, Julissa G. Portillo, Anais Rico, Laura V. Ruiz, Genevie M. Serrano, Kalia M. Sheldon, Hiroki Terada, Victoria A. Trujillo, Clarissa Vazquez-Ramos, Frank Wang, Dawn Flora, Felipe G. Zavala, Hartnell College Genomics Group

**Affiliations:** Division of Mathematics, Science, and Engineering, Hartnell College, Salinas, CA, USA

**Keywords:** Aphididae, *Chaetosiphon fragaefolii*, *Chaetosiphon thomasi*, mitogenome, strawberry aphid

## Abstract

The aphid *Chaetosiphon fragaefolii* Cockerell, 1901 is an agricultural pest and known vector of strawberry viruses. To better understand its biology and systematics, we performed a genomic analysis on *C. fragaefolii* collected from Quinalt strawberry plants from Pacific Grove, Monterey county, California, USA using Oxford Nanopore and Illumina sequencing. The resulting data were used to assemble the aphids complete mitogenome. The mitogenome of *C. fragaefolii* is 16,108 bp in length and contains 2 rRNA, 13 protein-coding, and 22 tRNA genes (GenBank accession number LC590896). The mitogenome is similar in content and organization to other Aphididae. Phylogenetic analysis of the *C. fragaefolii* mitogenome resolved it in a fully supported clade in the tribe Macrosiphini. Analysis of the *cox1* barcode sequence of *C. fragaefolii* from California found exact and nearly identical sequences to *C. fragaefolii* and *Chaetosiphon thomasi* Hille Ris Lambers, 1953, suggesting the two species are conspecific.

The Aphididae consists of more than 4700 species of aphids that occur worldwide (Kim and Lee 2008). About half of the species in the family are classified to its most specious subfamily, the Aphidinae (Choi et al. 2018). One of these species is the strawberry aphid, *Chaetosiphon fragaefolii* (Cockerell 1901). *C. fragaefolii* was originally named from specimens from Jerome, Arizona, but has since been reported throughout North and South America, Europe, South Africa, New Zealand, and Australia (Dixon et al. 1987; Blackman and Eastop 2000; Rondon and Cantliffe 2004). It is an agricultural pest and has been shown to transmit several viruses to strawberry plants, including the economically devastating strawberry mild yellow edge virus (Lavandero et al. 2012). A large number of *C. fragaefolii cox1* barcode gene sequences are deposited in GenBank (Foottit et al. 2008; Gwiazdowski et al. 2015; Hebert et al. 2016), however, the mitochondrial genome of *C. fragaefolii* has not been analyzed. Here, we performed Oxford Nanopore and Illumina genome sequencing on a specimen of *C. fragaefolii* from California, USA to determine its mitogenome structure and phylogenetic relationship to other aphids in the Aphididae.

DNA was extracted from *C. fragaefolii* (Voucher Specimen- Hartnell College #264, Dr. Jeffery R. Hughey, jhughey@hartnell.edu) collected on a Quinalt strawberry plant from Pacific Grove, Monterey county, California (36°37′06.1′′N, 121°54′41.1′′W) using the DNeasy Blood and Tissue Kit (Qiagen, Valencia, CA) following the protocol of Hughey et al. (2019). The DNA extract was concentrated to 10 µL using the Microcon DNA Fast Flow Centrifugal Filter Unit Cat # MRCF0R100 (MilliporeSigma, Burlington, MA). The Oxford Nanopore library and sequencing was performed using the Rapid Sequencing Kit (SQK-RAD004) on a R9.4.1 flow cell and MinION device following the manufacturer’s instructions (Oxford Nanopore Technologies, Oxford, UK). The Nanopore sequencing generated 161,000 reads. The 150 bp paired-end Illumina library construction and sequencing were performed by myGenomics, LLC (Alpharetta, GA) and generated 20,128,502 reads. The mitogenome was assembled *de novo* using Illumina reads with the default settings in MEGAHIT (Li et al. 2015), and the gaps closed by mapping both the Oxford Nanopore and Illumina reads onto the *de novo* contigs using the default settings in Geneious Prime^®^ 2020.1.2 (Biomatters Limited, Auckland, New Zealand). The annotation was completed with MITOS (Bernt et al. 2013) and NCBI ORF-finder (https://www.ncbi.nlm.nih.gov/orffinder/). The *C. fragaefolii* mitogenome was aligned to other mitogenomes with MAFFT (Katoh and Standley 2013) and the phylogenetic analysis was executed using RaxML in Trex-online (Boc et al. 2012) with the GTR + gamma model and 1,000 bootstraps. The tree was visualized with TreeDyn 198.3 at Phylogeny.fr (Dereeper et al. 2008).

The mitogenome of *C. fragaefolii* is 16,108 bp in length and is AT skewed with a base composition of 45.6% A, 38.3% T, 10.5% C, and 5.6% G. It contains 22 tRNA (*trnL* and *trnS* are duplicated), 2 rRNA (*rnl*, *rns*), and 13 electron transport and oxidative phosphorylation genes. Nine of the protein-coding genes and 15 tRNAs are coded on the forward strand, with the remaining 13 genes transcribed on the reverse strand. The start codon for the protein-coding genes *cox2*, *cox3*, *nad2*, and *nad6* is ATA; *nad1*, *nad4*, *nad4L*, and *nad5* is TTA; *cox1*, *atp6*, and *nad3* is ATT; *atp8* is ATC; and *Cyt b* is ATG. Most of the protein-coding genes terminate with TAA, but *nad4* and *nad4L* terminate with CAT; *cox*1 with ATT; and *nad1* with AAT. The mitogenome content and organization of *C. fragaefolii* is similar to other genera in the tribe Macrosiphini, including *Cavariella salicicola* (Wang et al. 2013), *Diuraphis noxia* (Zhang et al. 2014), *Indomegoura indica* (Hong et al. 2019), *Myzus persicae* (Voronova et al. 2020), and *Sitobion avenae* (Zhang et al. 2016). These taxa however differed from *Acyrthosiphon pisum* (International Aphid Genomics Consortium 2010) in the position of one of the *trnS* genes.

Phylogenetic analysis of *C. fragaefolii* fully resolved it in a clade with six other mitogenomes from the Macrosiphini (Figure 1). Comparison of the *C. fragaefolii cox1* California sequence to published *cox1* barcodes in GenBank found two identical sequences identified as *C. thomasi* from Manitoba and Saskatchewan, Canada. *Chaetosiphon thomasi* is a holocyclic rose-feeding species that specifically colonizes the rose plant *Rosa rugosa* (Blackman et al. 1987). The rose feeding populations of *C. thomasi* differ from *C. fragaefolii* in having a shorter rostrum and distinctive fundatrix morphology (Blackman et al. 1987). Thirty-one other sequences deposited in GenBank identified as *C. thomasi*, *C. fragaefolii*, and *Chaetosiphon* sp. from around the world differed by a single transition from adenine to guanine at position 246 of the *cox1* gene. The mutation is silent and codes for a methionine at amino acid 82 of *cox1*. Based on this genetic evidence, *C. fragaefolii* and *C. thomasi* appear to be conspecific, however both require DNA sequencing of topotype material before proposing a final taxonomic conclusion.

**Figure 1.**
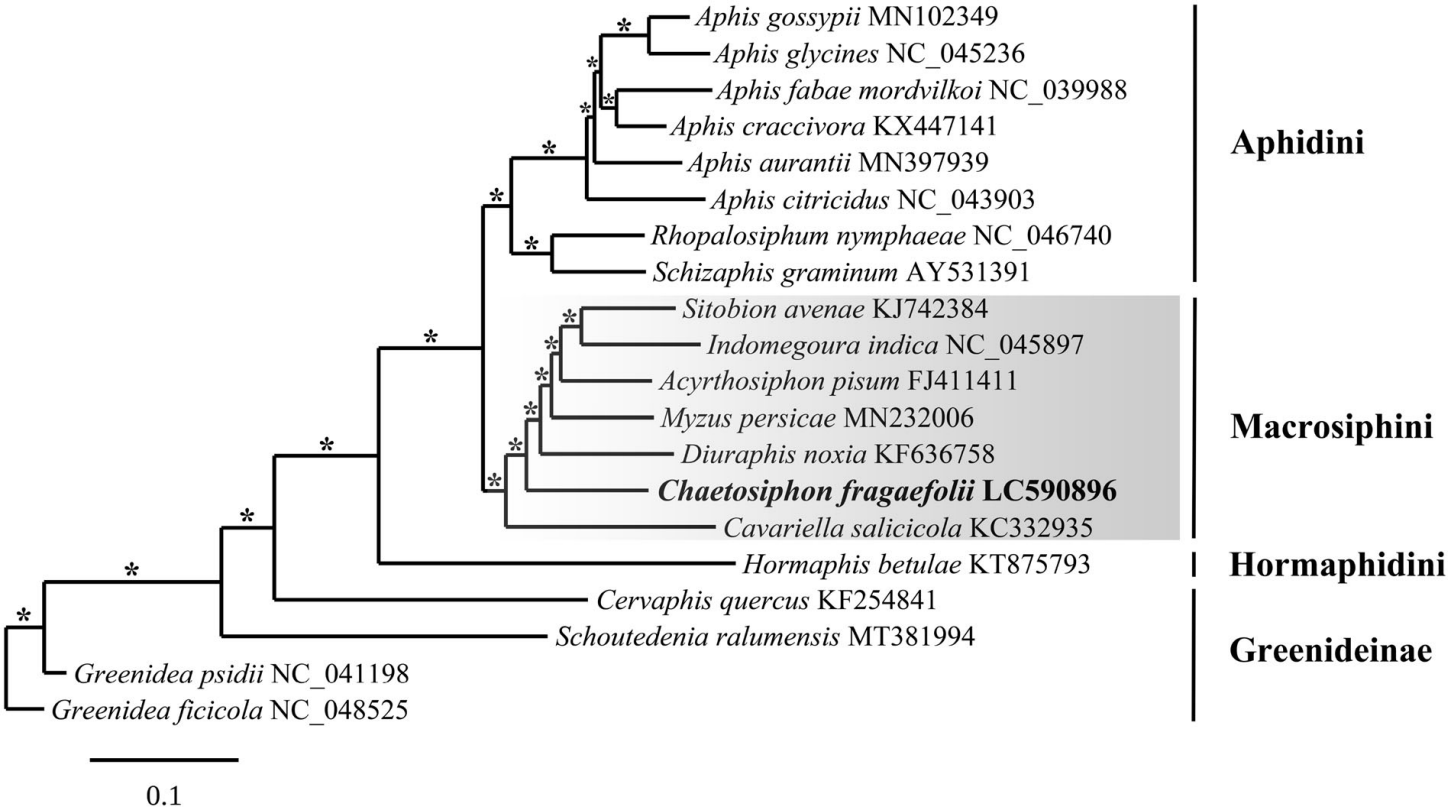
RaxML phylogram of *Chaetosiphon fragaefolii* and representative Aphididae mitogenomes. The subfamily Greenideinae served as the outgroup and the three other taxa listed to the right are tribes in the subfamily Aphidinae (Hormaphidini, Macrosiphini, and Aphidini). The * indicates 100% bootstrap support based on 1000 nreps. The legend below represents the scale for nucleotide substitutions.

## Data Availability

Mitogenome data supporting this study are openly available in GenBank at nucleotide database, https://www.ncbi.nlm.nih.gov/nuccore/LC590896, Associated BioProject, https://www.ncbi.nlm.nih.gov/bioproject/PRJNA666686, BioSample accession number at https://www.ncbi.nlm.nih.gov/biosample/SAMN16320332 and Sequence Read Archive at https://www.ncbi.nlm.nih.gov/sra/SRR12749480 and https://www.ncbi.nlm.nih.gov/sra/SRR12749481.

## References

[CIT0001] Bernt M, Donath A, Jühling F, Externbrink F, Florentz C, Fritzsch G, Pütz J, Middendorf M, Stadler PF. 2013. MITOS: improved de novo metazoan mitochondrial genome annotation. Mol Phylogenet Evol. 69(2):313–319. 22982435 10.1016/j.ympev.2012.08.023

[CIT0002] Blackman RL, Eastop VF. 2000. Aphids on the world’s crops: an identification and information guide. 2nd ed. New York (NY): Wiley; p. 324.

[CIT0024] Blackman RL, Eastop VF, Frazer BD, Raworth DA. 1987. The strawberry aphid complex, Chaetosiphon (Pentatrichopus) spp. (Hemiptera: Aphididae): taxonomic significance of variations in karyotype, chaetotaxy and morphology. Bull Entomol Res. 77:201–212.

[CIT0003] Boc A, Diallo AB, Makarenkov V. 2012. T-REX: a web server for inferring, validating and visualizing phylogenetic trees and networks. Nucleic Acids Res. 40:W573–W579. 22675075 10.1093/nar/gks485 PMC3394261

[CIT0004] Choi H, Shin S, Jung S, Clarke DJ, Lee S. 2018. Molecular phylogeny of Macrosiphini (Hemiptera: Aphididae): an evolutionary hypothesis for the Pterocomma-group habitat adaptation. Mol Phylogenet Evol. 121:12–22. 29253532 10.1016/j.ympev.2017.12.021

[CIT0005] Cockerell TDA. 1901. A new plant-louse injuring strawberry plants in Arizona. Can Entomol. 33(4):101–101.

[CIT0006] Dereeper A, Guignon V, Blanc G, Audic S, Buffet S, Chevenet F, Dufayard JF, Guindon S, Lefort V, Lescot M, et al. 2008. Phylogeny.fr: robust phylogenetic analysis for the non-specialist. Nucleic Acids Res. 36:W465–W469. 18424797 10.1093/nar/gkn180 PMC2447785

[CIT0007] Dixon AFG, Kindlmann P, Leps J, Holman J. 1987. Why there are so few species of aphids, especially in the tropics. Am Nat. 129(4):580–592.

[CIT0008] Foottit RG, Maw HE, Von Dohlen CD, Hebert PD. 2008. Species identification of aphids (Insecta: Hemiptera: Aphididae) through DNA barcodes. Mol Ecol Resour. 8(6):1189–1201. 21586006 10.1111/j.1755-0998.2008.02297.x

[CIT0009] Gwiazdowski RA, Foottit RG, Maw HE, Hebert PD. 2015. The hemiptera (insecta) of Canada: constructing a reference library of DNA barcodes. PLOS One. 10(4):e0125635. 25923328 10.1371/journal.pone.0125635 PMC4414572

[CIT0010] Hebert PDN, Ratnasingham S, Zakharov EV, Telfer AC, Levesque-Beaudin V, Milton MA, Pedersen S, Jannetta P, deWaard JR. 2016. Counting animal species with DNA barcodes: Canadian insects. Phil Trans R Soc B. 371(1702):20150333. 27481785 10.1098/rstb.2015.0333 PMC4971185

[CIT0011] Hille Ris Lambers D. 1953. Contribution to a monograph of the Aphididae of Europe. Temminckia. 9:1–177.

[CIT0012] Hong B, Zhang F, Hu ZQ, Zhao HY. 2019. The complete mitochondrial genome of *Indomegoura indica* (Hemiptera: Aphididae). Mitochondrial DNA B Resour. 4(1):882–883.

[CIT0013] Hughey JR, Maggs CA, Mineur F, Jarvis C, Miller KA, Shabaka SH, Gabrielson PW. 2019. Genetic analysis of the Linnaean *Ulva lactuca* (Ulvales, Chlorophyta) holotype and related type specimens reveals name misapplications, unexpected origins, and new synonymies. J Phycol. 55(3):503–508. 30907438 10.1111/jpy.12860

[CIT0014] International Aphid Genomics Consortium. 2010. Genome sequence of the pea aphid *Acyrthosiphon pisum*. PLoS Biol. 8:e1000313. 20186266 10.1371/journal.pbio.1000313 PMC2826372

[CIT0015] Katoh K, Standley DM. 2013. MAFFT multiple sequence alignment software version 7: improvements in performance and usability. Mol Biol Evol. 30(4):772–780. 23329690 10.1093/molbev/mst010 PMC3603318

[CIT0016] Kim H, Lee S. 2008. A molecular phylogeny of the tribe Aphidini (Insecta: Hemiptera: Aphididae) based on the mitochondrial tRNA/COII, 12S/16S and the nuclear EF1α genes. Syst Entomol. 33(4):711–721.

[CIT0017] Lavandero B, Rojas P, Ramirez CC, Salazar M, Caligari PDS. 2012. Genetic structure of the aphid, *Chaetosiphon fragaefolii*, and its role as a vector of the Strawberry Yellow Edge Virus to a native strawberry, *Fragaria chiloensis* in Chile. J Insect Sci. 12:110. 23438175 10.1673/031.012.11001 PMC3605023

[CIT0018] Li D, Liu CM, Luo R, Sadakane K, Lam TW. 2015. MEGAHIT: an ultra-fast single-node solution for large and complex metagenomics assembly via succinct de Bruijn graph. Bioinformatics. 31(10):1674–1676. 25609793 10.1093/bioinformatics/btv033

[CIT0019] Rondon S, Cantliffe DJ. 2004. *Chaetosiphon fragaefolii* (Homoptera: Aphididae): a potential new pest in florida? Fla Entomol. 87(4):612–615.2.0.CO;2]

[CIT0020] Voronova NV, Levykina S, Warner D, Shulinski R, Bandarenka Y, Zhorov D. 2020. Characteristic and variability of five complete aphid mitochondrial genomes: *Aphis fabae mordvilkoi*, *Aphis craccivora*, *Myzus persicae*, *Therioaphis tenera* and *Appendiseta robiniae* (Hemiptera; Sternorrhyncha; Aphididae). Int J Biol Macromol. 149:187–206. 31917211 10.1016/j.ijbiomac.2019.12.276

[CIT0021] Wang Y, Huang XL, Qiao GX. 2013. Comparative analysis of mitochondrial genomes of five aphid species (Hemiptera: Aphididae) and phylogenetic implications. PLOS One. 8(10):e77511. 24147014 10.1371/journal.pone.0077511 PMC3798312

[CIT0022] Zhang B, Ma C, Edwards O, Fuller S, Kang L. 2014. The mitochondrial genome of the Russian wheat aphid *Diuraphis noxia*: large repetitive sequences between trnE and trnF in aphids. Gene. 533(1):253–260. 24095774 10.1016/j.gene.2013.09.064

[CIT0023] Zhang B, Zheng J, Liang L, Fuller S, Ma CS. 2016. The complete mitochondrial genome of *Sitobion avenae* (Hemiptera: Aphididae). Mitochondrial DNA Part A. 27(2):945–946. 10.3109/19401736.2014.926498 24919501

